# Acceptance of Telemedicine Compared to In-Person Consultation From the Providers' and Users’ Perspectives: Multicenter, Cross-Sectional Study in Dermatology

**DOI:** 10.2196/45384

**Published:** 2023-08-11

**Authors:** Lara Valeska Maul, Anna Sophie Jahn, Gustavo S P Pamplona, Markus Streit, Lorena Gantenbein, Simon Müller, Mia-Louise Nielsen, Christian Greis, Alexander A Navarini, Julia-Tatjana Maul

**Affiliations:** 1 Department of Dermatology University Hospital Basel Basel Switzerland; 2 Jules-Gonin Eye Hospital/Fondation Asile des Aveugles Department of Ophthalmology University of Lausanne Lausanne Switzerland; 3 Rehabilitation Engineering Laboratory (RELab) Department of Health Sciences and Technology ETH Zurich Zurich Switzerland; 4 Department of Dermatology Cantonal Hospital Aarau Aarau Switzerland; 5 Department of Dermatology Copenhagen University Hospital-Bispebjerg Copenhagen Denmark; 6 Department of Dermatology University Hospital Zurich Zurich Switzerland

**Keywords:** acceptance, patient, physician, satisfaction, teledermatology

## Abstract

**Background:**

Teledermatology is currently finding its place in modern health care worldwide as a rapidly evolving field.

**Objective:**

The aim of this study was to investigate the acceptance of teledermatology compared to in-person consultation from the perspective of patients and professionals.

**Methods:**

This multicenter, cross-sectional pilot study was performed at secondary and tertiary referral centers of dermatology in Switzerland from August 2019 to January 2020. A customized questionnaire addressing demographics and educational data, experience with telemedicine, and presumed willingness to replace in-patient consultations with teledermatology was completed by dermatological patients, dermatologists, and health care workers in dermatology.

**Results:**

Among a total of 664 participants, the ones with previous telemedicine experience (171/664, 25.8%) indicated a high level of overall experience with it (patients: 73/106, 68.9%, dermatologists: 6/8, 75.0%, and health care workers: 27/34, 79.4%). Patients, dermatologists, and health care workers were most likely willing to replace in-person consultations with teledermatology for minor health issues (353/512, 68.9%; 37/45, 82.2%; and 89/107, 83.2%, respectively). We observed a higher preference for telemedicine among individuals who have already used telemedicine (patients: *P<*.001, dermatologists: *P*=.03, and health care workers, *P*=.005), as well as among patients with higher educational levels (*P*=.003).

**Conclusions:**

This study indicates that the preference for teledermatology has a high potential to increase over time since previous experience with telemedicine and a higher level of education were associated with a higher willingness to replace in-patient consultations with telemedicine. We assume that minor skin problems are the most promising issue in teledermatology. Our findings emphasize the need for dermatologists to be actively involved in the transition to teledermatology.

**Trial Registration:**

ClinicalTrials.gov NCT04495036; https://classic.clinicaltrials.gov/ct2/show/NCT04495036

## Introduction

Teledermatology is finding its place in modern health care worldwide as a rapidly evolving field. Telemedicine is defined as the use of telecommunication technology to provide clinical health care from a distance [1]. Due to its visual character, dermatology is regarded as a particularly suitable telemedicine application [2-4]. The individual attitude toward teledermatology has favorably changed within the last 10 years, as reported in a recent survey of British dermatologists [5]. During the COVID-19 pandemic, remote consultations have markedly increased in importance [6]. However, a prerequisite for widespread adoption of telemedicine is that it must favorably compete with in-person consultations on several objective measures, including effectiveness [7,8], availability [9,10], and costs [11]. During the COVID-19 pandemic, video- and audio-only visits were reimbursed even at the same rate as face-to-face visits in the United States [12]. A current debate in the United States on whether telemedicine increases spending and whether it improves patient outcomes leads to an unclear future for telemedicine [12], whereas France extends its tele-expertise funding even after the pandemic [13].

Nowadays, teledermatology is accepted as a valid tool and gains popularity in many countries [2,14]. Of paramount importance is also the acceptance and overall experience among patients as users and physicians as providers. Thus, telemedical services can benefit from provider- and user-friendly adaptations to implement and further develop this growing medical sector. Also, the World Health Organization has recently recognized and accepted that telemedicine is an important tool to allow health care access in remote areas and underserved communities [15]. The worldwide use of teledermatology is highest in Europe and North America, whereas regions with poor geographical distributions of doctors appear to be underrepresented [2]. Various studies reveal high levels of user satisfaction in teledermatology over the last 2 years [14]. However, common barriers reported by dermatologists include low reimbursement, concerns about government regulations, and liability [16].

Various studies suggest that dermatologists may achieve comparable accuracy in diagnosis and management with teledermatology in comparison to classical in-person visits [17-22]. The target group benefiting most from teledermatology seems to be rather older, immobilized patients with chronic wounds, as well as patients with treatment monitoring of inflammatory and infectious dermatoses [1,19,23]. Teledermatology bears enormous potential for being less stressful and saving time and money, especially for immobilized patients living in rural regions far away from health care centers [1]. Certain prerequisites and concerns of telemedicine include the implementation of appropriate devices and the technical skills involved in their handling, data safety, and diagnostic reliability [5]. Further arising concerns are the lack of a patient-physician relationship due to the remote nature of the procedure and insufficient follow-up [24,25]. Anxiety about being photographed is another reported reason for patients’ dissatisfaction with teledermatology [26]. Patients’ acceptance seems to be often neglected in current studies addressing this topic [1].

For several decades now, various models have been developed and used to investigate the use and acceptance of IT [27]. The “technology acceptance model” (TAM) is still one of the most popular theories to analyze the perception and factors influencing the acceptance of a novel technology [28]. First developed by Davis in 1986, the central goal of TAM was to increase the usage of IT by promoting its acceptance [29]. Thus, knowledge of the factors that contribute to acceptance is essential for this purpose. After being validated several times, the TAM model has been improved and adapted in 2000 (TAM 2), focusing on the “perceived usefulness,” and 2008 (TAM 3), focusing on the “perceived ease of use.” Venkatesh et al [30] validated a new model called the “unified theory of acceptance and use of technologies” (UTAUT), which is based on the conceptual similarities of 8 different models. The UTAUT model states that the intention to use a technology can be divided into “performance expectancy,” “effort expectancy,” “social influence,” and “facilitating conditions” [30]. The use of the most recent model version, UTAUT2, has been validated in several studies and is an important theoretical approach [31-34].

Given the sparse current data about the combination of users’ and providers’ overall experience with teledermatology, the aim of this study was to investigate the acceptance of telemedicine from different perspectives. By focusing on 3 target groups (patients, physicians, and health care workers) and asking physicians and health care workers about their patients’ views for the first time, we identified a research gap in dermatology. Further, to identify the acuity and severity of skin problems for which patients, health care workers in dermatology, and dermatologists would be willing to replace in-person consultations with telemedicine, we analyzed individual differences influencing satisfaction with telemedicine. In this study, we intended to focus on the practical value for practitioners and health policy makers which is why we used study-specific questionnaires and not validated ones.

## Methods

### Study Design and Participating Population

This multicenter, cross-sectional study was conducted at 1 secondary and 2 tertiary referral centers for dermatology in Switzerland. We applied a customized questionnaire from August 1, 2019, to January 31, 2020, at the Department of Dermatology of the University Hospitals of Basel and Zurich and at the Cantonal Hospital of Aarau. The questionnaire was offered to all patients aged 18 years and older at check-in and anonymously completed in the waiting room before the appointment with the dermatologist at the mentioned centers. We also sent an adapted version of the questionnaire to all dermatologists and medical staff (nurses and secretaries) at these hospitals (henceforth referred to as “health care workers”) to be completed at any time. The questionnaire was also applied to other physicians who do not work in dermatology; however, these data were not included in this study. Overall, 70.6% (512/725) of the patients, 72.6% (45/62) of the dermatologists, and 68.6% (107/156) of the health care workers responded to the questionnaire. Exclusion criteria were language barriers, cognitive impairment, and a lack of informed consent.

### Study Procedures and Questionnaire

We designed the questionnaire in German to assess individual characteristics and telemedicine-related aspects in patients (see questionnaire Q-A in Multimedia Appendix 1), as well as in physicians and health care workers (see questionnaire Q-B in Multimedia Appendix 2). A total of 12 questionnaire items were identical across cohorts. The questionnaire addressed demographics, educational, and economic data (currency was converted from Swiss Francs to Euros [1.0 CHF=1.0 Euro, July 4, 2022]), as well as aspects of individual experience with and opinion about telemedicine. Here, we only assessed the presumed willingness to replace in-person consultation and the overall experience with telemedicine, as well as their associations with individual characteristics. The 4 different categories of acuity and severity (minor, severe, acute, and chronic) were defined as the subjective, individual judgment of each participant for the acuity and severity of skin diseases. Other aspects of the questionnaire will be published later. Depending on the question, possible answers were either binary (yes/no), multiple choice, visual analog scales (VASs) with scores from 0-10, or free text.

### Data Analysis

We reported the proportions of primary answers and investigated the differences in the questionnaire answers related to the previous use of telemedicine (Q-A #12a, Q-B #5), additional in-person consultation to the telemedicine (Q-A #15, Q-B #8), media for telemedicine (Q-A #14, Q-B #7), overall experience with telemedicine consultation (Q-A #16, Q-B #9), presumed willingness to replace in-person consultation with telemedicine (Q-A #19, Q-B #11), preference for telemedicine or in-person consultation (Q-A #17-18, Q-B #10), and individual differences (Q-A #0-2, Q-B #0-2) across the 3 different cohorts (here defined as patients, physicians, and health care workers). Differences in proportion of answers across cohorts were explored with chi-square tests, with Monte-Carlo resampling with 10,000 iterations if the frequency in any cell was less than 5. Differences in answers for presumed willingness to replace in-person consultation with telemedicine across cohorts were evaluated for different severities (minor and severe) and acuities (chronic and acute) of skin problems. Differences in answers for preference for telemedicine or in-person consultation across individual differences were evaluated for each cohort separately. Differences in age were explored with the Kruskal-Wallis test. *P* values were adjusted for multiple comparisons using the false discovery rate (FDR) for each analysis separately. All analyses were performed through R (versions 3.6.2 and 4.2.0; The R Foundation) and Python version 3.7.6 (Python Software Foundation).

### Ethics Approval

The study was approved by the Northwest and Central Switzerland Ethics Committee (2019-00523) and was registered with ClinicalTrials.gov (NCT04495036). The study was conducted in full compliance with the Declaration of Helsinki (1964) and Good Clinical Practice was maintained throughout the study.

## Results

### Patient Demographics

A total of 512 dermatological patients (mean age 49.5, SD 17.9 years; 239/512, 46.7% women), 45 dermatologists (mean age 34.1 SD 8.5 years; 32/45, 71.1% women) and 107 health care workers (mean age 38.6, SD 13.0 years; 88/107, 82.2% women) completed the questionnaire (a total of 664 individuals). Information about nationality, place of residence, monthly salary, highest level of education, work experience, and previous use of telemedical services for patients, physicians, and health care workers is summarized in Table 1.

**Table 1:** Characteristics and telemedicine-related aspects of the study population (N=664). “A currency exchange rate of Euro €1=US $1.093 is applicable.”

Characteristics		Patients (n=512), n (%)	Dermatologists (n=45), n (%)		Health care workers (n=107), n (%)
**Gender**					
	Female	239 (46.7)	32 (71.1)		88 (82.2)
	Male	268 (52.3)	13 (28.9)		18 (16.8)
	N/A^a^	5 (1)	0 (0)		1 (0.9)
**Nationality**					
	Swiss	425 (83)	27 (60.0)		84 (78.5)
	Other	84 (16.4)	17 (37.8)		21 (19.6)
	N/A	3 (0.6)	1 (2.2)		2 (1.9)
**Place of residence**					
	Urban (>100,000 inhabitants)	88 (17.2)	35 (77.8)		30 (28)
	Urban (10,000-100,000 inhabitants)	146 (28.5)	5 (11.1)		26 (24.3)
	Rural (<10,000 inhabitants)	278 (54.3)	5 (11.1)		50 (46.7)
	N/A	0 (0)	0 (0)		1 (0.9)
**Monthly salary (in Euros)**					
	≤2000	59 (11.5)	N/A		N/A
	2000-5000	164 (32)	N/A		N/A
	5000-8000	136 (26.6)	N/A		N/A
	≥8000	97 (18.9)	N/A		N/A
	N/A	56 (10.9)	N/A		N/A
**Highest level of education**					
	Primary and secondary school	52 (10.2)	N/A	N/A	
	Apprenticeship	204 (39.8)	N/A	N/A	
	High school diploma	69 (13.5)	N/A	N/A	
	University or college degree	166 (32.4)	N/A	N/A	
	N/A	21 (4.1)	N/A	N/A	
**Medical occupation**					
	Resident in dermatology	N/A	29 (64.4)		N/A
	Board certification in dermatology	N/A	16 (35.6)		N/A
**Work experience (years)**					
	≤5	N/A	19 (42.2)		25 (23.4)
	5-10	N/A	17 (37.8)		22 (20.1)
	10-20	N/A	4 (8.9)		24 (22.4)
	20-30	N/A	4 (8.9)		21 (19.6)
	≥30	N/A	1 (2.2)		12 (11.2)
	N/A	N/A	0 (0)		3 (2.8)
**Already used telemedicine (as a patient)?**					
	Yes	123 (24)	9 (20)		39 (36.4)
	No	382 (74.6)	36 (80)		68 (63.6)
	N/A	7 (1.4)	0 (0)		0 (0)
**Already used teledermatology?**					
	Yes	23 (18.7)	N/A		N/A
	No	92 (74.8)	N/A		N/A
	N/A	8 (6.5)	N/A		N/A
**Already worked as a teledermatology provider?**					
	Yes	N/A	19 (29.7)		4 (3.7)
	No	N/A	45 (70.3)		73 (68.2)
	N/A	N/A	0 (0)		30 (28)
**Health insurance based on telemedicine**					
	Yes	64 (12.5)	6 (13.3)		17 (15.9)
	No	51 (10)	3 (6.7)		20 (18.7)
	N/A	397 (77.5)	36 (80)		70 (65.4)
**Daily internet use (in hours)**					
	≤1	209 (40.8)	7 (15.6)		39 (36.4)
	1-2	160 (31.2)	22 (48.9)		36 (33.6)
	2-3	58 (11.3)	9 (20)		19 (17.8)
	3-4	26 (5.1)	3 (6.7)		7 (6.5)
	>4	38 (7.4)	4 (8.9)		3 (2.8)
	N/A	21 (4.1)	0 (0)		3 (2.8)

^a^N/A: not applicable or did not answer.

### Overall Experience With Telemedicine Consultation

Considering only individuals who reported having already used telemedicine (henceforth referred to as “telemedicine users”; Q-A #12a, Q-B #5, Table 1) and having an in-person consultation for the same medical problem (Q-A #15, Q-B #8), telemedicine was used before rather than after an in-person consultation by 84.4% (54/64) of the patients, 83.3% (5/6) of the dermatologists, 92.3% (24/26) of the health care workers and we observed no differences across cohorts (*χ*²=1.1; *P*=.70).

Considering only previous telemedicine users (Q-A #12a, Q-B #5, Table 1), the overall experience with telemedicine from patients’ perspective within all 3 target group was rated as either “very good” or “good” by 68.9% (73/106) of the patients, 75.0% (6/8) of the physicians, and by 79.4% (27/34) of the health care workers (Q-A #16, Q-B #9, Table 2) and we observed no differences across cohorts (*χ*²=7.3; *P*=.50, Table 2), excluding the “not applicable/did not answer” responses. We also report information about media used for telemedicine counselling in Table S1 in Multimedia Appendix 3 (Q-A #14, Q-B #7).

**Table 2:** Overall experience with use of telemedicine from the patients’ perspective within the 3 target groups. The *P* value did not include the “not applicable/did not answer” responses.

Rating	Patients’ perspective (n=120), n (%)	Patients’ perspective within dermatologists (n=9), n (%)	Patients’ perspective within health care workers (n=39), n (%)	*P* value
Very good	18 (15)	2 (22.2)	6 (15.4)	.50
Good	55 (45.8)	4 (44.4)	21 (53.8)	.50
Regular	25 (20.8)	1 (11.1)	6 (15.4)	.50
Bad	6 (5)	0 (0)	1 (2.6)	.50
Very bad	2 (1.7)	1 (11.1)	0 (0)	.50
N/A^a^	14 (11.7)	1 (11.1)	5 (12.8)	.50

^a^N/A: not applicable or did not answer.

### Presumed Willingness to Replace In-Person Consultation With Telemedicine for Different Acuities and Severities of Skin Problems

Considering all respondents, they reported that they could consider replacing the in-person consultation with telemedicine for minor skin problems (patients: 353/512, 68.9%; dermatologists: 37/45, 82.2%; and health care workers: 89/107, 83.2%), but not for severe (patients: 30/512, 5.9%; dermatologists: 4/45, 8.9%; and health care workers: 7/107, 6.5%), acute (patients: 122/512, 23.8%; dermatologists: 22/45, 48.9%; and health care workers: 39/107, 36.4%), and chronic skin problems (patients: 120/512, 23.4%; dermatologists: 19/45, 42.2%; and health care workers: 37/107, 34.6%; Q-A #19, Q-B #11, Table 3).

**Figure 1:**
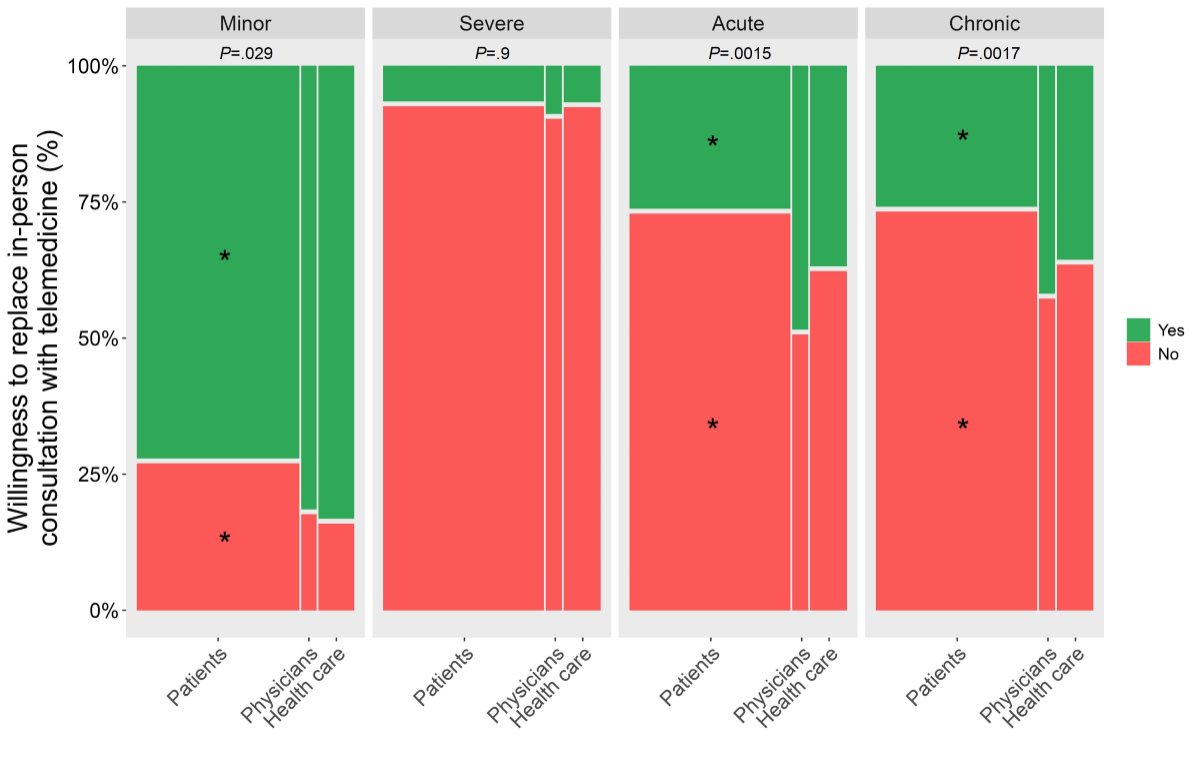
Presumed willingness to replace in-person consultation with telemedicine across cohorts for different severities and acuities of skin problem. Green and red areas represent the presumed willingness to replace in-person consultation with telemedicine (“yes”) or not (“no”), respectively. *P* values for the chi-square tests are given on top of the plots. * Asterisks represent significant pairwise comparisons in post-hoc analyses (*P*<.05) following chi-square tests, corrected for multiple comparisons using the false discovery rate (FDR) method.

We observed differences across cohorts in the responses when individuals were asked if they could consider replacing in-person consultation with telemedicine for minor (*χ*²_2_=7.09; *P*=.04), acute (*χ*²_2_=12.95; *P*=.008), and chronic (*χ*²_2_=8.12; *P*=.03) skin problems, but not for severe skin problems. Nonresponses (not applicable or did not answer) have not been included in statistical tests (Q-A #19, Q-B #11, Table 3, Figure 1). Post hoc analyses revealed a lower presumed willingness to replace in-person consultation with telemedicine for minor and chronic skin problems among patients compared to other cohorts. Furthermore, post hoc analyses also revealed a higher presumed willingness to replace in-person consultation with telemedicine for acute skin problems among physicians compared to other cohorts.

**Table 3:** Presumed willingness to replace in-person consultation with telemedicine across cohorts for different severities and acuities of skin problems. Nonresponses were excluded from statistical tests.

Severity and acuity of skin problem and willingness to replace in-person consultations with telemedicine		Patients (n=512), n (%)	Dermatologists (n=45), n (%)	Health care workers (n=107), n (%)	*P* value
**Minor problem**					.04
	No	132 (25.8)	8 (17.8)	17 (15.9)	
	Yes	353 (68.9)	37 (82.2)	89 (83.2)	
	N/A^a^	27 (5.3)	0 (0)	1 (0.9)	
**Severe problem**					.90
	No	427 (83.4)	41 (91.1)	97 (90.7)	
	Yes	30 (5.9)	4 (8.9)	7 (6.5)	
	N/A	55 (10.7)	0 (0)	3 (2.8)	
**Acute problem**					.008
	No	339 (66.2)	23 (51.1)	66 (61.7)	
	Yes	122 (23.8)	22 (48.9)	39 (36.4)	
	N/A	51 (10.0)	0 (0)	2 (1.9)	
**Chronic problem**					.03
	No	340 (66.4)	26 (57.8)	66 (61.7)	
	Yes	120 (23.4)	19 (42.2)	37 (34.6)	
	N/A	52 (10.2)	0 (0)	4 (3.7)	

^a^N/A: not applicable or did not answer.

### Preference for Telemedicine or In-Person Consultation

Considering all respondents (ie, including individuals with no previous experience with telemedicine), the reported preference as a patient for telemedicine or in-person consultation (Q-A #17, Q-B #10) across skin problems and several individual characteristics for all cohorts is shown in Table S2 in Multimedia Appendix 3.

**Figure 2:**
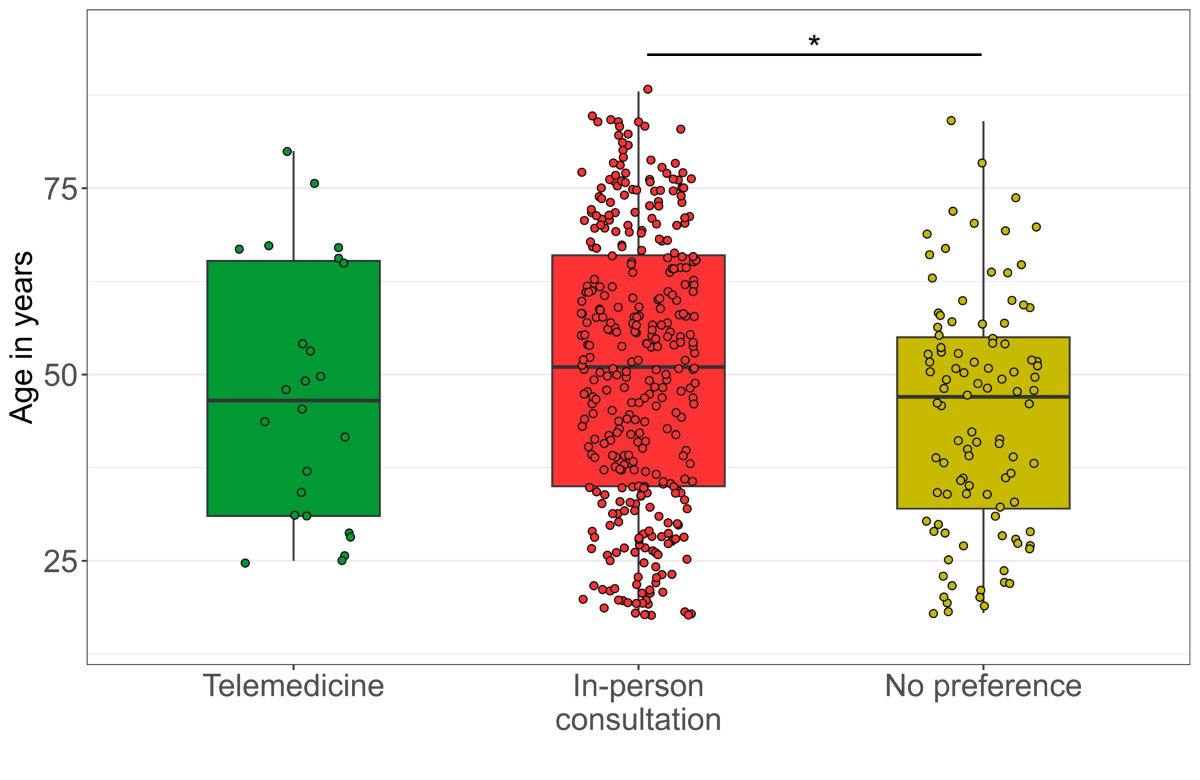
Preference for telemedicine or consultation across patients’ age. Patients’ preference for in-person consultation is associated with higher age compared to individuals who reported no preference, with no difference in the preference between in-person consultation and telemedicine. The asterisk represents a significant difference following posthoc analysis, corrected for multiple comparisons using the false discovery rate (FDR) method.

**Figure 3:**
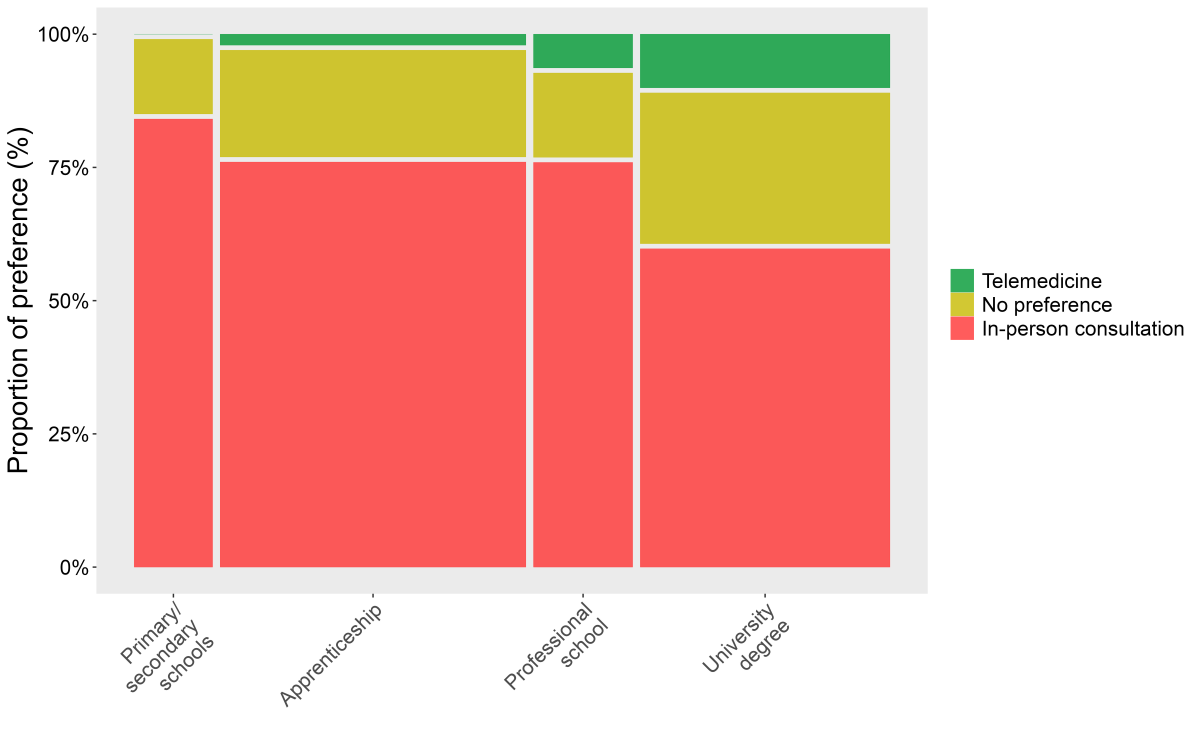
Association between patients’ highest education level achieved and preference for telemedicine or in-person consultation: higher preference for telemedicine among patients with university degree. Asterisks represent significant pairwise comparisons following posthoc analysis, corrected for multiple comparisons using the false discovery rate (FDR) method.

Analyzing the cohorts separately, we observed differences in preference for telemedicine or in-person consultation (or no preference) with patient age (Q-A #0.1; *P*=.01) and physician age (Q-B #0.1; *P*=.02) (Table S2 in Multimedia Appendix 3, Figure 2). Post hoc analysis showed that the age of those who reported preferring in-person consultation was higher than the age of those who reported no preference, with no difference between in-person consultation and telemedicine. In addition, we observed that patients’ preferences for telemedicine or in-person consultation were different across education levels (*χ*²=24.6; *P*<.001; Q-A #0.5, Figure 3). Post hoc analysis indicated a higher preference for telemedicine in patients with university degrees compared to other levels of education.

**Figure 4:**
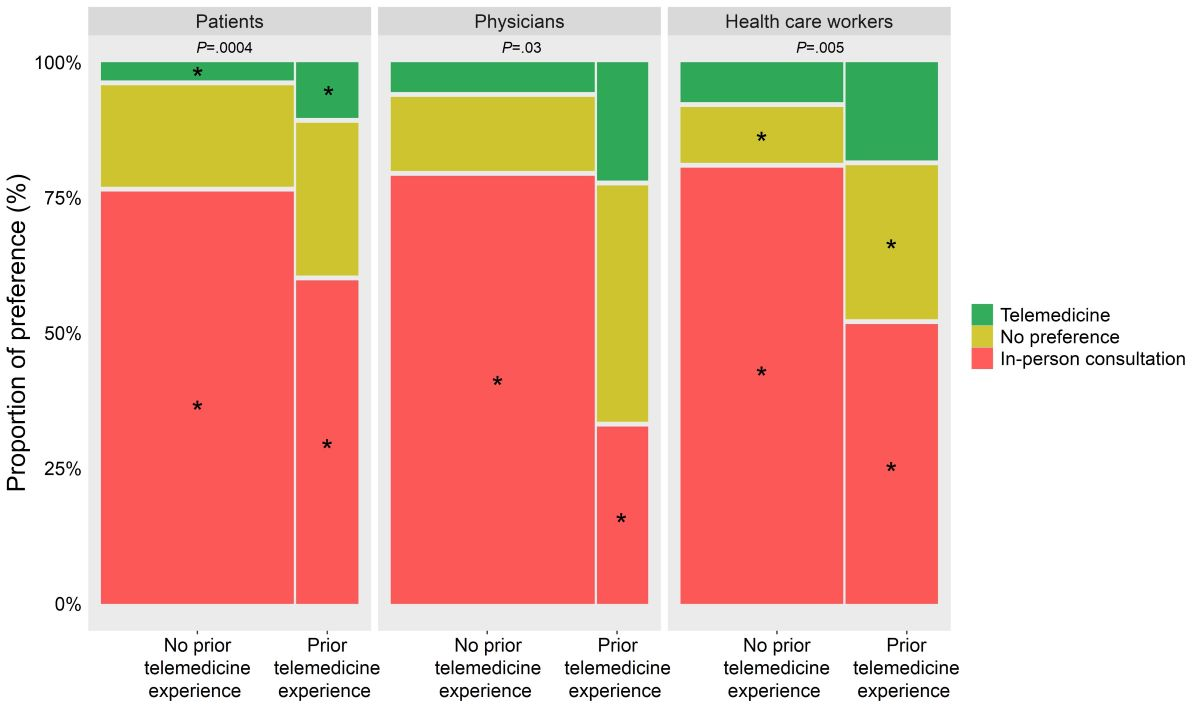
Higher preference for telemedicine over in-person consultation depended on previous telemedicine experience of patients, physicians, and health care workers. *P* values for chi-square tests are shown on top of the plots. Asterisks represent significant pairwise comparisons in posthoc analyses, corrected for multiple comparisons using the false discovery rate (FDR) method.

Furthermore, we observed that the preference of patients (*χ*²_2_=15.6; *P*<.001), dermatologists (*χ*²_2_=7.87; *P*=.03), and health care workers (*χ*²=10.3; *P*=.005) for telemedicine depended on their previous telemedicine experience (Q-A #12a, Q-B #5, Figure 4). Post hoc analyses indicated that, with previous telemedicine experience, there is a higher preference for telemedicine and lower preference for in-person consultation among patients; a higher preference for in-person consultation among physicians; and a higher reporting of no preference and lower preference for in-person consultation among health care workers. We observed no other differences in preference for telemedicine for the other considered individual characteristics, for either the cohorts analyzed separately (Table S2 in Multimedia Appendix 3) or for patients currently using telemedicine (Table S3 in Multimedia Appendix 3).

## Discussion

### Overview

Understanding the population’s current attitudes toward telemedicine is highly relevant to optimizing the transition to teledermatology. We found that individuals who have previously used telemedicine reported a high level of overall experience with it. Patients, health care workers, and physicians reported that they would consider replacing in-person consultations with telemedicine for minor health issues. We observed a higher preference for telemedicine among patients with higher levels of education and among individuals who had previous experience with telemedicine.

### Overall Experience With Telemedicine Consultation

This study revealed high overall experience among all individuals with their use of telemedicine (between 68.9% and 79.4%), which is consistent with previous studies [35-37]. However, satisfaction with telemedicine is controversial. In a comparative study with 121 patients, only 44% of them reported satisfaction with telemedicine, while 10% of them reported dissatisfaction [38]. Despite the relevance of better understanding the perspectives of medical staff and nonmedical staff, this study is the first to examine the physicians and health care workers’ level of overall experience with telemedicine from their perspective as patients, to the best of our knowledge.

The COVID-19 pandemic has triggered a new era in telemedicine with an immense increase in applications within various medical fields [39,40], as telemedicine has minimized the hazard of direct exposure to individuals during the pandemic. A Brazilian study identified that the posture toward telemedicine of physicians as providers was positively influenced and significantly increased from 18.5% before the pandemic to 63.6% during the COVID-19 period [41]. Furthermore, over 70% of patients at New York University Langone Health reported being satisfied with live-interactive teledermatology during the pandemic in 2020 [42]. Our findings indicate that the overall experience with telemedicine in the Swiss-German population was already comparably high before the pandemic. We further postulate that our findings on the level of overall experience should now be even higher than reported here, as pandemic conditions have further strengthened telemedicine services.

### Presumed Willingness to Replace In-Person Consultation With Telemedicine for Different Acuities and Severities of Skin Problems

In the context of teledermatology, we found that the presumed willingness to replace in-person consultation with telemedicine depends on the severity and acuity of the skin problem. Most patients, physicians, and health care workers favored replacement for minor skin problems. We observed that, among the cohorts analyzed, the highest level of acceptance for telemedicine for acute and chronic skin problems was among physicians. Most participants would not consider replacing in-person consultations with telemedicine for severe skin issues.

Previous studies have suggested that in-person consultations cannot generally be replaced by telemedicine [43,44]. The identification of the most suitable indications for telehealth versus traditional consultations is crucial for maintaining patients’ safety and satisfaction. The COVID-19 pandemic has transformed the health care system through a sudden expansion of telemedicine for nonurgent and urgent care [40]. Data from the pandemic period showed an increase of 683% in teledermatology usage in the United States, particularly for urgent care [45]. Even during the pandemic, the patients preferred in-person consultations over telemedicine for severe conditions [45], which is consistent with our pre–COVID-19 findings.

On the other hand, a systematic review indicated that, for particular situations, telemedicine can adequately replace in-person consultation [46]. The willingness to use telemedicine after the pandemic was reported by many studies [46]. One study reported that only a minority of patients (36%) consider that telemedicine is not appropriate to replace in-person consultations [47]. We suggest that teledermatology services should take into account the acuity and severity of skin problems in the future and thereby focus on the diagnoses best handled with telemedicine.

### Preference for Telemedicine or In-Person Consultation

Our data indicate that patients, physicians, and health care workers with experience in telemedicine and patients with a higher educational level showed a higher preference for telemedicine. As expected, older patients and physicians favored in-person consultations.

Our findings imply that one of the main problems telemedicine is facing is trust among new patients. We observed that a first experience with telemedicine influences one’s attitude toward it positively. As we detected that there is somehow a barrier to the first use of telemedicine, some acclimatization needs to occur [48]. In a previous study with 184 teledermatology patients, telemedicine experience was also associated with a higher odds ratio of preferring teledermatology in the future [49]. Also, during the COVID-19 pandemic, first-time teledermatology patients in the United States reported being less satisfied with telemedicine communication [36].

Furthermore, we also observed that a high preference for telemedicine was associated with a higher educational level. We suggest that the finding is related to the increased attitude of highly educated individuals to inform themselves about new technologies and to have sufficient technical skills [50].

This study indicates that older patients and physicians prefer in-person consultations over telemedicine. An American study with >600 participants reported that patients older than 66 years of age preferred in-person consultation and follow-up during the COVID-19 pandemic [42]. In fact, another study indicated higher satisfaction rates with telemedicine among patients younger than 56 years of age and without suspected cancer [51]. While studies focusing only on teledermatology generally report higher levels of satisfaction, direct comparative studies of in-person consultations and telemedicine report mixed outcomes [26,51-56]. Consistent with our findings, a questionnaire-based survey among 720 patients in Saudi Arabia during the COVID-19 pandemic showed that older age (over 40 years), lower education levels, and first-time experience were associated with poor-to-average satisfaction with telemedicine [57].

We identified factors associated with a lower preference for teledermatology: older age, lower education, and no experience with telemedicine. As teledermatology becomes more prominent, these cohorts may develop disparities in care. Therefore, we propose that the following steps should be considered to increase acceptance of teledermatology: minimization of technical barriers by simple handling of the telemedical offers as well as increasing the attractiveness of the first telemedicine consultation through reduced waiting time and cost savings.

Despite technological improvements, such as increased access to mobile phones and high-resolution screening, the latest studies about satisfaction with teledermatology revealed that patients and dermatologists still prefer in-person examinations [51,58]. We speculate that this reluctance to use telemedicine may be related to accessibility skills and previous knowledge about telemedicine. Future assessments could be designed to guide us in handling teledermatology as a screening tool.

The era of telemedicine has also opened new horizons for rapid digital professional exchange of expertise between physicians of different specialties and the ability to obtain a second opinion [13,59,60]. Tele-expertise is cost-effective as patients can reduce doctor -patient contact with a specialist [61]. Remote areas and the pandemic have taught us that tele-expertise alongside teledermatology is of high importance because access to primary care is limited, especially in these scenarios [62]. Future studies focusing on the challenges, integration, and quality of tele-expertise in relation to telemedicine are needed.

### Limitations

This study has some limitations. First, there was a low number of dermatologists and health care workers since we predefined the centers. This is also the reason why sample size was not beforehand determined, but we aimed to maximize the sampling given our time and site predefinitions. While this study might be minimally representative of the dermatologists’ perspective in German-speaking Switzerland working at massive health care centers, it may be difficult to generalize the findings to a larger population of medical professionals. We suggest that data for dermatologists should be viewed with caution when interpreting the emerging patterns. Second, our findings may not be generalizable outside of Switzerland. However, as our results were consistent with related literature, we speculate that it is likely that our findings can be reproduced in similar cultures. Third, the results might have been influenced by confounders, such as dermatologists being mostly women and living in large cities. Thus, we cannot really conclude if the differences are due to their gender, profession, and place of residence. Further research assessing the acceptance of teledermatology in a larger study population, especially also in countries with a geographically challenging health care situation and including the distinction between live-interactive and store-and-forward applications will help to better understand the impending value of telemedicine. Due to a lack of usage of nonvalidated scales to measure the variables, our results’ value might be less important in scientific research but has a higher impact on clinical daily routines and health policy makers.

### Conclusions

This study indicates that acceptance and preference for teledermatology have a high potential to increase over time as they depend on experience with telemedicine. Acclimatization is needed for new users and providers. We assume that minor skin problems are the most promising field for teledermatology, and thus we suggest focusing on them for further development. This study indicated that within the 3 target groups, 65.4% to 80% of individuals were overchallenged with the question of whether their insurance covers telemedicine services. This finding provides a relevant insight for health policy makers and health insurance managers. The reimbursement of telemedicine services is not yet regulated evenly within countries and globally which reflects the ongoing debate of its acceptance. There is a high need to expand telemedicine and teledermatology in order to provide access to medicine and care for patients globally. We provide new insights into the telemedicine situation in German-speaking Switzerland and emphasize the need for dermatologists to be actively involved in the transition to teledermatology. Future studies integrating well-established models such as UTAUT2 are required to focus on an in-depth view of the different target groups after the outbreak of the pandemic.

## Appendix

Multimedia Appendix 1 Supplementary Figure 1: Questionnaire about telemedicine for patients in dermatology (Q-A).

Multimedia Appendix 2 Supplementary Figure 2: Questionnaire about telemedicine for healthcare workers in dermatology and dermatologists (Q-B).

Multimedia Appendix 3 Supplemantary Tables: (Tables S1-3).

## Abbreviations

FDR: false discovery rate
TAM: technology acceptance model
UTAUT: unified theory of acceptance and use of technologies
VAS: visual analogue scale
