# Supplementary material for: Acceptance of Telemedicine Compared to In-Person Consultation From the Providers' and Users’ Perspectives: Multicenter, Cross-Sectional Study in Dermatology
Source: JMIR Dermatol. 2023 Aug 11;6:e45384. doi: 10.2196/45384 (PMC10457706; doi:10.2196/45384)
Supplement: Multimedia Appendix 2 [file derma_v6i1e45384_app2.docx]

**Survey on telemedicine for**

**healthcare workers in dermatology and dermatologists**

**Thank you for your participation in this anonymous survey!**

To evaluate the current use and acceptance of telemedicine in German-speaking Switzerland

(University and cantonal hospitals), we are very interested in your opinion.

***What is telemedicine?***

*Telemedicine is medicine over distance (spatially and possibly also temporally). The doctor and patient do not sit face to face, but are connected via telephone, internet, video and/or app.*

**First, we would like to ask you some general questions.**

**0.1 Age in years:** *_________* **0.2 female male**

**0.3 Nationality:**  Swiss others:___

**0.4 Profession:**

Medical staff (e.g. nurses, medical secretaries)

Resident in dermatology

Board certification in dermatology

Resident in other specialty: ______________

Board certification in other specialty: __________________

**1. How many years of professional experience do you have since your exam?**

< 5 years 5-10 years 10-20 years 20-30 years > 30 years

**2. Do you live in a city or in the countryside?**

urban (>100.000 inhabitants) urban (>10.000-100.000) rural

**3. How much time do you spend for one day online/in the internet?**

≤ 1 hour ≤ 2 hours ≤ 3 hours ≤ 4 hours > 4 hours

**4. How do you access the internet?**

Smartphone Tablet Laptop Computer due to others (e.g. grandchild)

**Now we'd like to ask you some questions about telemedicine for your own health problems.**

**5. Have you ever used telemedical services for yourself as a patient?**

yes no

***If you have NOT had any personal contact with telemedical services, please continue with number 10.***

**6. Do you have a telemedical insurance?**

yes no

**7. Which telemedical services have you used so far as a patient?**

Internet consultation via chat / e-mail

Telephone consultation

Online video consultation

App consultation via smartphone

Name of the company: ___________________________________________

**8. Have you also consulted a doctor personally for the same medical problem?**

BEFORE online consultation AFTER online consultation no

**9. What was your overall experience with telemedical consultation?**

very good good moderate bad very bad

***___________________________________________________________________________________***

**10.** **Would you *– as a patient yourself -* generally prefer to visit a doctor in person or would you prefer an online doctor's appointment (telemedicine)?**

Telemedicine Face to face consultation No preference

**11. Can you currently imagine replacing the personal doctor's appointment with telemedicine for the following medical questions:**

- **for minor skin problems? a)**  yes no
- **for severe skin problems? b)**  yes no
- **for acute skin problems? c)**  yes no
- **for chronic skin problems? d)**  yes no

**12. What is the maximum waiting time you would accept for a telemedical appointment?**

none 10 minutes 60 minutes 24 hours 1 week

**13. What is the maximum amount you would be willing to spend on telemedical consultation?**

50 CHF 75 CHF 100 CHF 200 CHF > 200 CHF

**14. a) Would you pay a surcharge for an immediate online doctor's appointment available on demand (telemedicine without waiting time)?**

yes no

**b) If yes: What is the maximum surcharge you would pay for this?**

10 CHF 25 CHF 50 CHF 100 CHF > 100 CHF

**Now we would like to ask you some questions about telemedicine from a professional perspective as a healthcare professional or dermatologists.**

**15. What advantages do you see in a personal visit to the doctor? (You can choose more than one answer here).**

Better diagnosis through direct physical examination

Better assessment of the doctor's competence through a personal interview

Personal relationship with the doctor

Higher data security of my medical record

Easier implementation compared to telemedicine

I feel an overall improvement in my quality of life through the personal visit to the doctor.

Others: _­­­­­­­­­­­­­­­­­­­­­­­___________________________________________

**16.** **What advantages do you see for yourself in telemedicine**? (You can choose more than one answer here)

Time saving

Cost saving

Lesser / no waiting time

multilingual consultancy

Privacy by anonymous contact

Access to medical care regardless of location

I feel an overall improvement in the quality of life through telemedicine.

others: __________________________________________________

**17. What disadvantages do you see for yourself in telemedicine**? (You can choose more than one answer here)

Lack of personal doctor-patient relationship

Questionable data security with online connection

Source of error in diagnosis due to spatial distance

Anonymity

High technical effort

Lack of confidence

No clear benefits, no improvement in quality of life

Others: _____ *_____________________________________________*

**18. How should the telemedical consultation ideally be structured from your point of view?**

Contact via chat or e-mail

Contact via chat or e-mail with photo of the physician

Contact via telephone

Contact via video conference

Combination of written, telephone and visual contact

**19. Would you like to be able to select the gender and/or location of the doctor in this process?**

No

Gender selection option

Possibility to choose the location of the doctor (domestic or abroad)

**20. In your opinion, for which skin diseases is telemedicine suitable?** (You can choose more than one answer here)

Inflammatory skin diseases (e.g. psoriasis, neurodermatitis, eczema)

Acne / Rosacea

Acute skin diseases (e.g. acute wounds, rash, hives)

Chronic wounds (ulcers)

Skin cancer screening

Skin cancer

Sexual transmitted disease

Itching

Skin diseases in children

Cosmetic skin problem

Others: ___________________________________________________________

**21.** **How do you assess the quality of telemedical consultation in principle?**

very good good moderate bad very bad

**22. How do you rate the credibility of telemedicine consultation compared to in-person consultation?**

significantly better rather better equivalent slightly worse significantly worse

**23. How high do you currently estimate the use of telemedicine by dermatological patients in Switzerland?**

< 10% 10-25% 25-50% 50-75% 75-90% > 90%

**24. Have you already provided telemedicine consultations yourself as a physician?**

yes no

**25. Can you imagine being active in telemedicine in the future?**

yes no
