# Supplementary material for: Acceptance of Telemedicine Compared to In-Person Consultation From the Providers' and Users’ Perspectives: Multicenter, Cross-Sectional Study in Dermatology
Source: JMIR Dermatol. 2023 Aug 11;6:e45384. doi: 10.2196/45384 (PMC10457706; doi:10.2196/45384)
Supplement: Multimedia Appendix 3 [file derma_v6i1e45384_app3.docx]

**Supplementary Tables**

**Table S1. Media for previous use of telemedicine**

| Medium for telemedicine consultation | Variable | Patients (n = 123) | Physicians (n = 11) | Healthcare workers (n = 39) | p-values |
| --- | --- | --- | --- | --- | --- |
| Internet | Yes | 12.2% (15) | 18.2% (2) | 12.8% (5) | 0.94 |
|  | No | 77.2% (95) | 81.8% (9) | 87.2% (34) |  |
|  | NA | 10.6% (13) | 0% (0) | 0% (0) |  |
| Phone | Yes | 78.9% (97) | 100% (11) | 82.1% (32) | 0.93 |
|  | No | 9.7% (12) | 0% (0) | 17.9% (7) |  |
|  | NA | 11.4% (14) | 0% (0) | 0% (0) |  |
| Video call | Yes | 0% (0) | 0% (0) | 0% (0) | NA |
|  | No | 89.4% (110) | 100% (11) | 100% (39) |  |
|  | NA | 10.6% (13) | 0% (0) | 0% (0) |  |
| Phone app | Yes | 5.7% (7) | 0% (0) | 7.7% (3) | 0.94 |
|  | No | 82.9% (102) | 100% (11) | 92.3% (36) |  |
|  | NA | 11.4% (14) | 0% (0) | 0% (0) |  |

*Note:* NA = not applicable/did not answer. Values between parentheses correspond to the number of individuals.

**Table S2. Preference for telemedicine or in-person consultation across cohorts for factors describing individual characteristics**

| Cohort | Individual characteristic | Variable | Preference for telemedicine | Preference for in-person consultation | No preference | p-values |
| --- | --- | --- | --- | --- | --- | --- |
| Patients | Median age in years [IQR] |  | 46.5 [31.0, 65.2] | 51.0 [35.0, 66.0] | 47.0 [32.0, 55.0] | .01 |
|  | Gender | Female | 45.8% (11) | 47.3% (164) | 48.5% (49) | 1 |
|  |  | Male | 54.2% (13) | 52.7% (183) | 51.5% (52) |  |
|  | Nationality | Swiss | 87.5% (21) | 83.9% (292) | 86.3% (88) | .9 |
|  |  | Other | 12.5% (3) | 16.1% (56) | 13.7% (14) |  |
|  | Highest level of education | Elementary and high school | 0 | 12.6% (42) | 6.9% (7) | .003 |
|  |  | Apprenticeship | 16.7% (4) | 44.4% (148) | 38.6% (39) |  |
|  |  | Professional school | 16.7% (4) | 14.4% (48) | 9.9% (10) |  |
|  |  | University degree | 166.7% (16) | 28.5% (95) | 44.6% (45) |  |
|  | Monthly salary | ≤ Euro 2’000 | 8.7% (2) | 14.2% (44) | 10.9% (10) | .4 |
|  |  | Euro 2’000 – 5000 | 17.4% (4) | 38.5% (119) | 32.6% (30) |  |
|  |  | Euro 5’000 – 8’000 | 39.1% (9) | 27.8% (86) | 28.3% (26) |  |
|  |  | > Euro ‘8000 | 34.8% (8) | 19.4% (60) | 28.3% (26) |  |
|  | Place of residence | Urban (> 100’000 inhabitants) | 20.8% (5) | 15.7% (55) | 19.6% (20) | .8 |
|  |  | Urban (10’000 – 100’000 inhabitants) | 37.5% (9) | 28.2% (99) | 28.4% (29) |  |
|  |  | Rural  (< 10’000 inhabitants) | 41.7% (10) | 56.1% (197) | 52.0% (53) |  |
|  | Previous use of telemedicine | No | 50.0% (12) | 79.8% (277) | 67.3% (68) | .003 |
|  |  | Yes | 50.0% (12) | 20.2% (70) | 32.7% (33) |  |
|  | Previous use of teledermatology | No | 63.6% (7) | 80.3% (53) | 84.4% (27) | .5 |
|  |  | Yes | 36.4% (4) | 19.7% (13) | 15.6% (5) |  |
| Dermatologists | Median age in years [IQR] |  | 31.0 [30.7, 31.7] | 33.5 [30.5, 39.2] | 30.0 [25.0, 31.0] | .02 |
|  | Gender | Female | 75.0% (3) | 68.7% (22) | 77.8% (7) | .9 |
|  |  | Male | 25.0% (1) | 31.2% (10) | 22.2% (2) |  |
|  | Nationality | Swiss | 75.0% (3) | 61.3% (19) | 55.6% (5) | .9 |
|  |  | Other | 25.0% (1) | 38.7% (12) | 44.4% (4) |  |
|  | Place of residence | Urban (> 100’000 inhabitants) | 100% (4) | 75.0% (24) | 77.8% (7) | .4 |
|  |  | Urban (10’000 – 100’000 inhabitants) | 0 | 9.4% (3) | 22.2% (2) |  |
|  |  | Rural  (< 10’000 inhabitants) | 0 | 15.6% (5) | 0 |  |
|  | Telemedicine provider | No | 50.0% (2) | 71.9% (23) | 55.6% (5) | .5 |
|  |  | Yes | 50.0% (2) | 28.1% (9) | 44.4% (4) |  |
|  | Previous use of telemedicine | No | 50.0% (2) | 90.6% (29) | 55.6% (5) | .03 |
|  |  | Yes | 50.0% (2) | 9.4% (3) | 44.4% (4) |  |
| Healthcare workers | Median age in years [IQR] |  | 42.5 [35.2, 48.8] | 36.0 [27.0, 50.0] | 35.5 [27.2, 42.8] | .9 |
|  | Gender | Female | 91.7% (11) | 81.1% (60) | 94.4% (17) | .6 |
|  |  | Male | 8.3% (1) | 18.9% (14) | 5.6% (1) |  |
|  | Nationality | Swiss | 83.3% (10) | 75.3% (55) | 94.4% (17) | .6 |
|  |  | Other | 16.7% (2) | 24.7% (18) | 5.6% (1) |  |
|  | Place of residence | Urban (>  100’000 inhabitants) | 25.0% (3) | 31.1% (23) | 22.2% (4) | .9 |
|  |  | Urban (10’000 – 100’000 inhabitants) | 25.0% (3) | 21.6% (16) | 33.3% (6) |  |
|  |  | Rural  (< 10’000 inhabitants) | 50.0% (6) | 47.3% (35) | 44.4% (8) |  |
|  | Telemedicine provider | No | 83.3% (5) | 98.2% (54) | 85.7% (12) | .9 |
|  |  | Yes | 16.7% (1) | 1.8% (1)) | 14.3% (2) |  |
|  | Previous use of telemedicine | No | 41.7% (5) | 73.3% (55) | 38.9% (7) | .03 |
|  |  | Yes | 58.3% (7) | 26.7% (20) | 61.1% (11) |  |

*Note:* Asterisks represent significant differences across cohorts (p < 0.05), corrected for multiple comparisons using the FDR method. IQR = interquartile range. Values between parentheses correspond to the number of individuals.

**Table S3. Preference for telemedicine or in-person consultation in patients currently consulting for skin problems across factors describing individual characteristics**

| Individual characteristic | Variable | Preference for telemedicine | Preference for in-person consultation | No preference | p-values |
| --- | --- | --- | --- | --- | --- |
| Median age in years [IQR] |  | 44.0 [31.0, 46.5] | 51.0 [35.0, 64.0] | 49.0 [33.5, 55.5] | 0.6 |
| Gender | Female | 63.6% (7) | 46.9% (206) | 47.5% (19) | 0.9 |
|  | Male | 36.4% (4) | 53.1% (233) | 52.5% (21) |  |
| Nationality | Swiss | 90.9% (10) | 84.1% (370) | 80.5% (33) | 0.9 |
|  | Other | 9.1% (1) | 15.9% (70) | 19.5% (8) |  |
| Highest level of education | Elementary and high school | 0 | 10.6% (45) | 7.7% (3) | 0.4 |
|  | Apprenticeship | 18.2% (2) | 42.9% (182) | 35.9% (14) |  |
|  | Professional school | 0 | 13.9% (59) | 17.9% (7) |  |
|  | University degree | 81.8% (9) | 32.5% (138) | 38.5% (15) |  |
| Monthly salary | < CHF 2 000 | 27.3% (3) | 13.2% (52) | 10.8% (4) | 0.9 |
|  | CHF 2 000 – 5 000 | 9.1% (1) | 36.3% (143) | 35.1% (13) |  |
|  | CHF 5 000 – 8 000 | 36.4% (4) | 28.9% (114) | 32.4% (12) |  |
|  | > CHF 8 000 | 27.3% (3) | 21.6% (85) | 21.6% (8) |  |
| Place of residence | Urban (> 100 000 inhabitants) | 9.1% (1) | 17.4% (77) | 17.1% (7) | 0.9 |
|  | Urban (10 000 – 100 000 inhabitants) | 27.3% (3) | 27.8% (123) | 31.7% (13) |  |
|  | Rural  (< 10 000 inhabitants) | 63.6% (7) | 54.9% (243) | 51.2% (21) |  |
| Median quality of life impairment [IQR] |  | 3.0 [0.0, 4.5] | 2.0 [0.0, 5.0] | 2.0 [0.0, 4.0] | 0.9 |
| Previous use of telemedicine | No | 63.6% (7) | 76.7% (335) | 68.3% (28) | 0.9 |
|  | Yes | 36.4% (4) | 23.3% (102) | 31.7% (13) |  |
| Previous use of teledermatology | No | 75.0% (3) | 82.3% (79) | 72.7% (8) | 0.9 |
|  | Yes | 25.0% (1) | 17.7% (17) | 27.3% (3) |  |

*Note:* IQR = interquartile range. Values between parentheses correspond to the number of individuals.
